# Chitin Nanofibrils and Nanolignin as Functional Agents in Skin Regeneration

**DOI:** 10.3390/ijms20112669

**Published:** 2019-05-30

**Authors:** Serena Danti, Luisa Trombi, Alessandra Fusco, Bahareh Azimi, Andrea Lazzeri, Pierfrancesco Morganti, Maria-Beatrice Coltelli, Giovanna Donnarumma

**Affiliations:** 1 Department of Civil and Industrial Engineering, University of Pisa, 56122 Pisa, Italy; andrea.lazzeri@unipi.it (A.L.), maria.beatrice.coltelli@unipi.it (M.-B.C.); 2 Consorzio Interuniversitario Nazionale per la Scienza e Tecnologia dei Materiali (INSTM), 50121 Florence, Italy; l.trombi@yahoo.it (L.T.), alessandra.fusco@unicampania.it (A.F.), b.azimi@ing.unipi.it (B.A.), giovanna.donnarumma@unicampania.it (G.D.); 3 Department of Experimental Medicine, University of Campania “Luigi Vanvitelli”, 80138 Naples, Italy; pierfrancesco.morganti@mavicosmetics.it

**Keywords:** chitin, lignin, anti-inflammatory, immunomodulation, keratinocytes, mesenchymal stem cells, cosmetics, glycyrrhetinic acid, skin regeneration, bio-based, nanomaterials

## Abstract

Chitin and lignin, by-products of fishery and plant biomass, can be converted to innovative high value bio- and eco-compatible materials. On the nanoscale, high antibacterial, anti-inflammatory, cicatrizing and anti-aging activity is obtained by controlling their crystalline structure and purity. Moreover, electropositive chitin nanofibrlis (CN) can be combined with electronegative nanolignin (NL) leading to microcapsule-like systems suitable for entrapping both hydrophilic and lipophilic molecules. The aim of this study was to provide morphological, physico-chemical, thermogravimetric and biological characterization of CN, NL, and CN-NL complexes, which were also loaded with glycyrrhetinic acid (GA) as a model of a bioactive molecule. CN-NL and CN-NL/GA were thermally stable up to 114 °C and 127 °C, respectively. The compounds were administered to in vitro cultures of human keratinocytes (HaCaT cells) and human mesenchymal stromal cells (hMSCs) for potential use in skin contact applications. Cell viability, cytokine expression and effects on hMSC multipotency were studied. For each component, CN, NL, CN-NL and CN-NL/GA, non-toxic concentrations towards HaCaT cells were identified. In the keratinocyte model, the proinflammatory cytokines IL-1α, IL-1 β, IL-6, IL-8 and TNF-α that resulted were downregulated, whereas the antimicrobial peptide human β defensin-2 was upregulated by CN-LN. The hMSCs were viable, and the use of these complexes did not modify the osteo-differentiation capability of these cells. The obtained findings demonstrate that these biocomponents are cytocompatible, show anti-inflammatory activity and may serve for the delivery of biomolecules for skin care and regeneration.

## 1. Introduction

In recent years, the development of natural biopolymer-based products suitable for cosmetic, skin care and regeneration have received considerable attention [1]. The key specifications of these products are biocompatibility and biodegradation, both in the environment and in the human body. The constituent polymers and their decomposition products are thus expected to be safe and without side effects. Chitin and lignin, by-products of fishery and plant biomass, can be reused and converted to novel high value materials for biomedical and cosmetic applications, which are bio- and eco-compatible [2,3]. On the nanoscale, chitin and lignin crystalline structure and purity can be controlled, resulting in several interesting properties, such as antibacterial [4], anti-inflammatory [5], cicatrizing and anti-aging effectiveness [6,7]. Chitin nanofibrils (CN) represent the purest crystal form of chitin and show positive surface charges. As such, CN are able to combine with electronegative compounds entrapping different ingredients for skin-friendly applications, such as innovative cosmetics for aged [6,8], as well as problematic and sensitive skin [9]. Because of these properties, CN have been used in combination with biopolymers [3,10], such as with poly (lactic acid) (PLA) [11,12,13,14,15], by several researchers. Morganti et al. [6] developed CN-Hyaluronan block copolymeric nanoparticles, entrapping several active ingredients in order to evaluate their efficiency and safety as biologically active rejuvenation treatments able to accelerate skin regeneration. They concluded that these biodegradable polymer particles have some advantages over other colloidal carrier systems thanks to their higher stability and versatility in tailoring the ingredient load and its release rate. In another attempt [7], the same authors investigated CN-hyaluronan block-copolymeric nanoparticles for their ability to load lutein, as an active anti-wrinkle agent, and demonstrated their effectiveness as anti-aging cosmetic emulsion compounds. Combining electropositive CN and electronegative nanolignin (NL) also gives rise to microcapsules able to entrap both hydrophilic and lipophilic molecules such as vitamins, microelements, anti-inflammatory drugs, antioxidants, anti-ageing substances, immunomodulating agents and enzymes [3,16]. 

In view of the abovementioned first studies, since cosmetic product consumers are becoming more and more aware of the environmental impact of products and increasingly trust the efficacy of green ingredients, the interest in such biopolymeric nanomaterials is expected to grow in the coming years. Therefore, it is significantly important to assess the specific properties of CN and NL and CN-NL complexes, including their material characterization, in order to improve or enable efficient processing technologies and biological characterization, including usable concentrations and the disclosure of their advantages in skin contact products as well as potential side effects, if any. To this purpose, advanced in vitro platforms can be efficiently exploited as ethical models for material screening. The HaCaT cell line represents an established model of human keratinocytes (i.e., epidermis) which can give preliminary information about new materials with respect to their inflammatory reactions through the expression of several cytokines, and of antimicrobial response through the expression of antimicrobial peptides of the innate immunity, such as defensins. 

The aim of this study was to test in vitro the efficacy and safety of CN, NL and CN-NL complexes, obtained in powder by spray drying, with human keratinocytes (HaCaT cells) and human mesenchymal stromal cells (hMSCs). CN-LN was also investigated for the delivery of glycyrrhetinic acid (GA), as a biomolecule with anti-inflammatory activity deriving from licorice plants (Glycyrrhiza). GA (also known as enoxolone or glycyrrhetic acid) is a pentacyclic triterpenoid obtained from the hydrolysis of glycyrrhizic acid. GA and its salts and esters are cosmetic ingredients which act as flavoring or skin-conditioning agents and have excellent antimicrobial, anti-inflammatory, antioxidant, anti-ulceration, antiviral and analgesic properties [17,18]. In this context, hMSCs represent a model of immature cells present in the connective tissue (e.g., dermis), which may be affected by the addition of nanocomponents, as they can penetrate across skin layers. The hMSC multipotency (i.e., capability of multiple lineage differentiation proper of stem cells) must be preserved for a correct tissue turn over. 

To our knowledge, this is the first study investigating the efficacy of CN-NL complexes as carriers of GA. The successful fabrication of innovative complexes entrapping antibacterial and anti-inflammatory ingredients would enable the introduction of novel products and their industrial upscale for potential use in dermatologic and cosmetic applications. 

## 2. Results

### 2.1. Morphological Characterization of CN, NL and CN-NL Complexes

**Figure 1:**
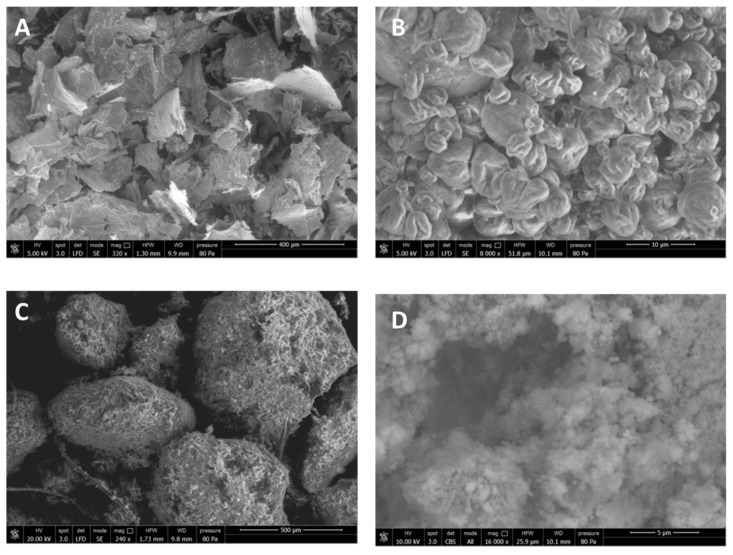
FE-SEM micrographs of the powders: (**A**) Spray dried CN; (**B**) Spray dried CN-NL complex; (**C**) Morphology of NL powder taken from the pristine NL sample; (**D**) Zoomed-in magnification of NL (16,000×) in back scattered modality to observe the NL nanostructure.

CN was spray-dried in the presence of 2% (*w*/*w*) of poly(ethylene glycol) (PEG) to obtain CN samples and consisted of scraps of micrometric dimensions (Figure 1A). The use of PEG can be advantageous to avoid CN aggregation when the solution is concentrated [15]. In fact, this aggregation, by giving rise to particles of micrometric size, would result in a lower effectiveness of CNs because of the reduction of their active surface. The spray-dried CN-NL complex in powder, pretreated with PEG, showed a specific morphology, as it consisted of almost round particles of micrometric dimensions (Figure 1B). The supplied lignin in powder, NL, appeared as nanostructured micrometric aggregates (Figure 1C,D).

**Figure 2:**
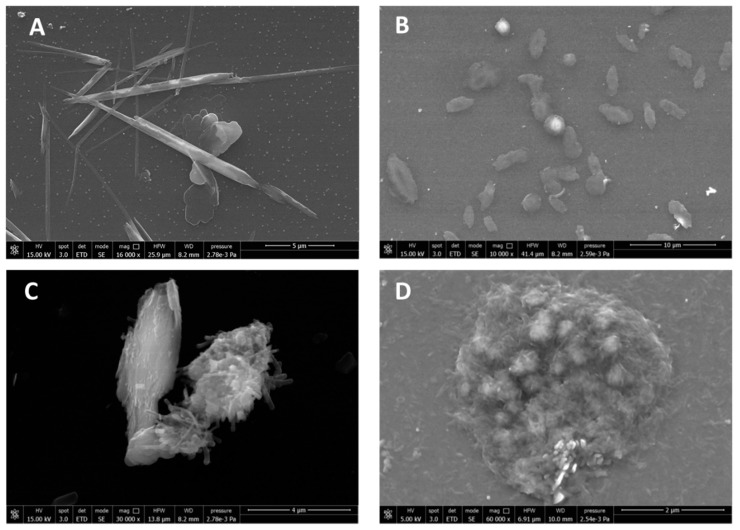
FE-SEM micrographs of (**A**) pure CN from deposition from diluted water suspension; (**B**) CN-NL complex; (**C**) and (**D**) CN-NL complex at higher magnification.

For better investigating the morphology of CN, pure CN suspensions at 2% (*w*/*w*) were diluted 1:1000 in distilled water and the suspension was deposited on a glass window on which the sample was dried. CN appeared as “whiskers” having a nanometric thickness and a micrometric length (Figure 2A). The spray dried complex CN-NL in powder (Figure 1B) was suspended in water (at 80 ppm) and analyzed after deposition on a glass window followed by drying. This sample showed a completely different morphology, consisting in micrometric disks having a round or ellipsoidal shape (Figure 2B). At higher magnification, the presence of a nanostructured system was highlighted (Figure 2C,D), in which the presence of both CN and NL particles could be observed. 

The spray-dried CN-NL complex was easily suspended in water to obtain flat micrometric nanostructured agglomerates that can deposit onto a surface, and be used to modify its properties thanks to CN and NL functionalities. 

### 2.2. Chemical Structure and Thermal Stability of CN, NL, GA and CN-NL/GA Complexes

**Figure 3:**
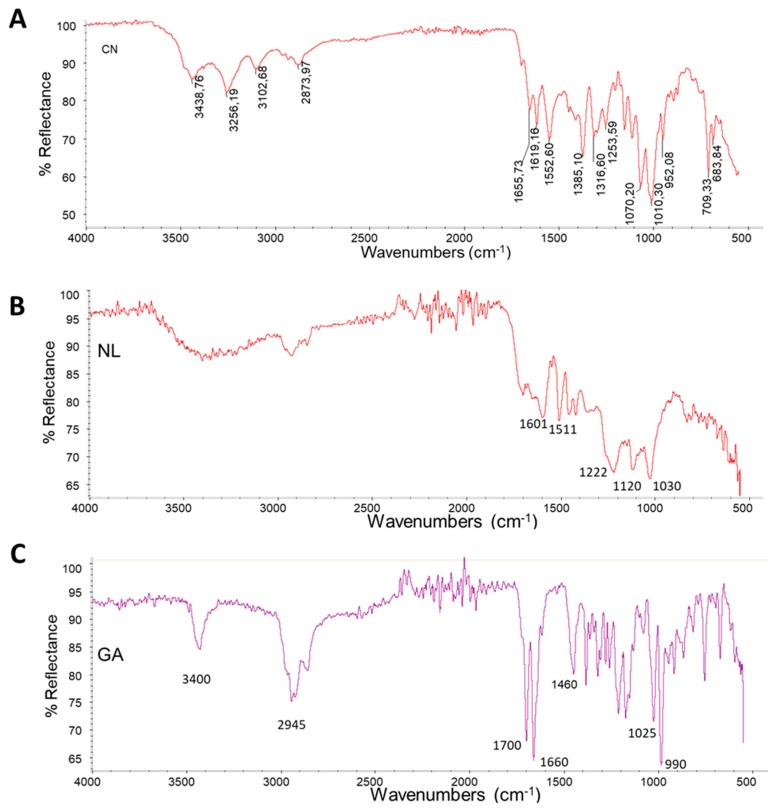
Infrared ATR spectra of (**A**): CN; (**B**) NL; (**C**) GA.

The starting materials and complexes CN-NL and CN-NL/GA were characterized by infrared spectroscopy. Spray-dried CN powder showed a spectrum (Figure 3A) with characteristic amide I and Amide II bands at 1619 and 1552 cm^−1^, respectively. The Amide I band is split into two components at 1656 cm^−1^ and 1619 cm^−1^. This is typical of α-chitin [19]. The most intense bands at 1010 cm^−1^ and 1070 cm^−1^ are typical of C-O stretching and agree with the polysaccharidic nature of this biopolymer. The band at 3439 cm^−1^ is attributable to O-H stretching, the bands at 3256 cm^−1^ and 3102 cm^−1^ to N-H stretching of amine and amide groups, respectively. The band at 2874 cm^−1^ indicates C-H stretching.

NL shows a spectrum (Figure 3B) characterized by the presence of strong band s at 1030 cm^−1^, 1120 cm^−1^ and 1222 cm^−1^. The band at 1222 cm^−1^ can be associated with C–C plus C–O plus C=O stretching.

The band at 1030 cm^−1^ can be attributed to aromatic C–H deformation that appears as a complex vibration associated with the C–O, C–C stretching and C–OH bending in polysaccharides. Carbohydrate originating vibrations are associated to the band at 1120 cm^−1^ [20]. In the carbonyl/carboxyl region, medium bands are found at 1705–1720 cm^−1^, attributable to unconjugated carbonyl/carboxyl stretching. The bands at 1601 cm^−1^ and 1511 cm^−1^ can be associated with aromatic skeleton vibrations. 

The GA spectrum (Figure 3C) is characterized by a band at 3430 cm^−1^ typical of OH stretching and a band at 2945 cm^−1^ attributable to CH stretching; both the bands at 1700 cm^−1^ and 1660 cm^−1^ are assigned to the C=O stretching (not conjugated and conjugated with the double bond, respectively) and the band at 1460 cm^−1^ to CH bending; the band at 1025 cm^−1^ can be attributed to C-O stretching and the band at 990 cm^−1^ can be attributed to the rocking of methyl groups [21].

**Figure 4:**
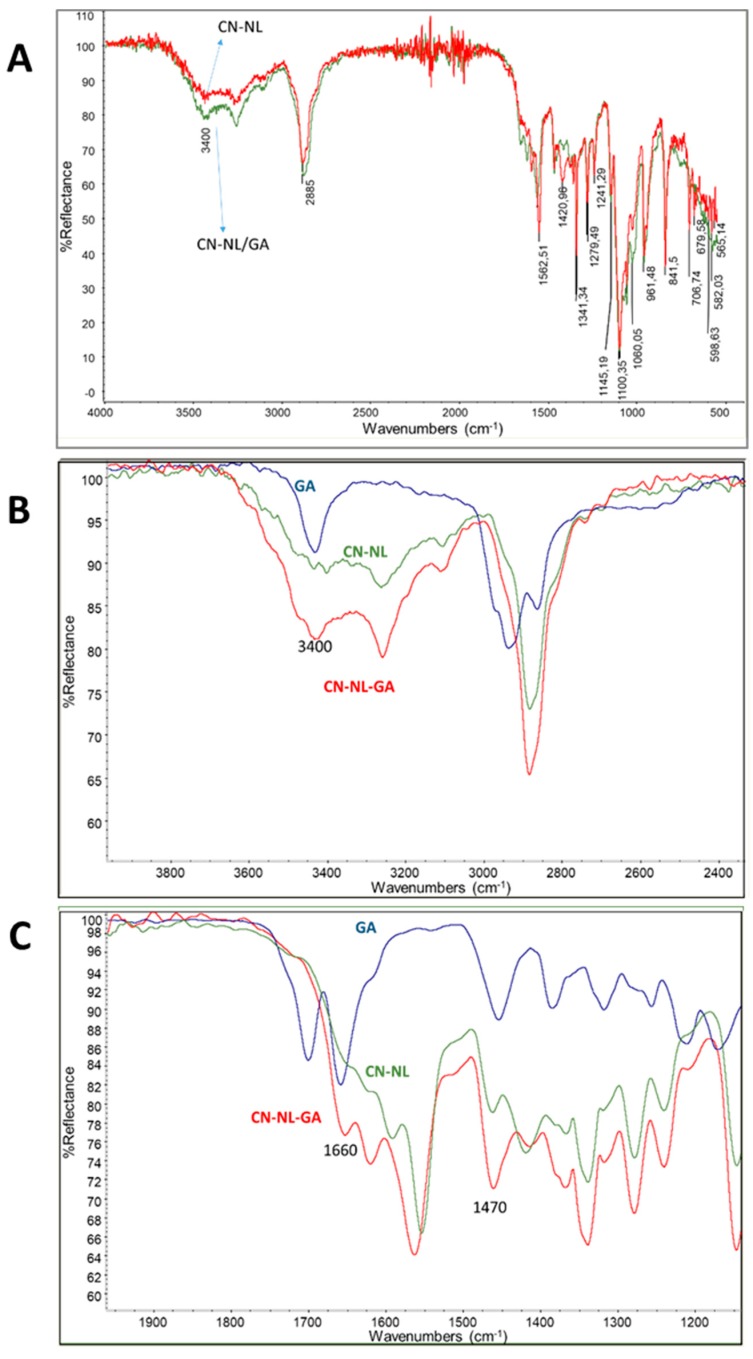
Infrared ATR spectra of (**A**) CN-NL and CN-NL/GA powders; (**B**) CN-NL, CN-LN/GA and GA in the region 2400-4000 cm^−1^; (**C**) CN-NL, CN-LN/GA and GA in the region 1100-2000 cm^-1.^

The complex CN-NL and the same complex entrapping GA were characterized by ATR spectroscopy (Figure 4). The obtained spectra were quite similar and they showed the bands already observed in the previous spectra of CN and NL, but slightly shifted because of the interactions occurring between CN and NL. The presence of GA can be revealed despite of its low concentration in the sample (0.2% w/w). As it can be observed in Figure 4B where the spectrum of GA, the spectrum of CN-NL and the spectrum of CN-NL/GA are compared in the region 2400–4000 cm^−1^, the higher intensity of the CN-NL/GA with respect to CN-NL at 3400 cm^−1^ is in agreement with the presence of GA in the CN-NL/GA complex. A similar comparison is proposed in Figure 4C for the region 1100-2000 cm^−1^. The increase of intensity of the band at 1650 cm^−1^ and 1470 cm^−1^ clearly observed in the spectrum of CN-NL/GA complex, can be attributed to the presence of GA in the powder. Interestingly the band at 1700 cm^−1^ of the carboxylic group of GA is not much evident in Figure 4C. In agreement with the work of Cheng et al. [22] this can be attributed to the interactions or reactions of the carboxylic groups with the functional group of CN and NL. 

**Figure 5:**
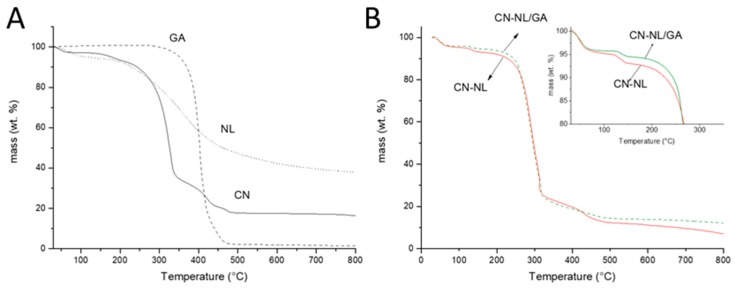
Thermogravimetric trends related to: (**A**) GA, NL and CN; (**B**) CN-NL and CN-NL/GA.

The thermogravimetric analysis (TGA) in nitrogen flow of pure components and of the two complexes was investigated and it was observed that GA, having an onset temperature at 387 °C, has a higher thermal stability than CN and NL. This different behavior is attributable to its action as a radical scavenger [23]. GA decomposition occurred in a single step and resulted in an almost complete decomposition. As observed in Figure 5A and in Table 1, the final residue is only 1.97% by weight. The behavior of CN and NL was different, as several mass loss steps were observed (Figure 5A) and the final residue was 38.9% for NL and 17.4% for CN. These results evidenced that NL and CN formed a high amount of carbonaceous residue during thermal decomposition. Interestingly, NL showed a not negligible mass loss at 40 °C, probably because of partial decomposition, resulting in volatile fragments.

The thermogravimetric trend of the complex did not include this mass loss. Hence, NL appeared more stable thanks to the formation of the complex with CN. However, the thermal stability of CN was reduced due to the presence of NL (the onset 1 and onset 2 temperatures were both decreased comparing those of CN-NL with CN) (Table 1). By the addition of GA, the stability of the complex was improved. This could be reasonably due to the radical scavenger activity of GA. Although the thermogravimetric trends of CN-NL and CN-NL/GA were similar, the mass loss recorded for CN-NL/GA was clearly lower than the one of CN-NL. This evidence corroborated the improved stability of CN-NL/GA thanks to the presence of GA.

### 2.3. HaCaT Cell Viability

HaCaT cells were seeded at a density of 1∙10^3^/well in 96-well culture plates. After 24 hours, the cells were treated with CN, CN-NL and CN-NL/GA at the following concentrations: 10 μg/mL, 5 μg/mL, μg/mL, 1 μg/mL, 0.5 μg/mL, 0.2 μg/mL, 0.1 μg/mL, 75 ng/mL, 50 ng/mL, 25 ng/mL.

**Figure 6:**
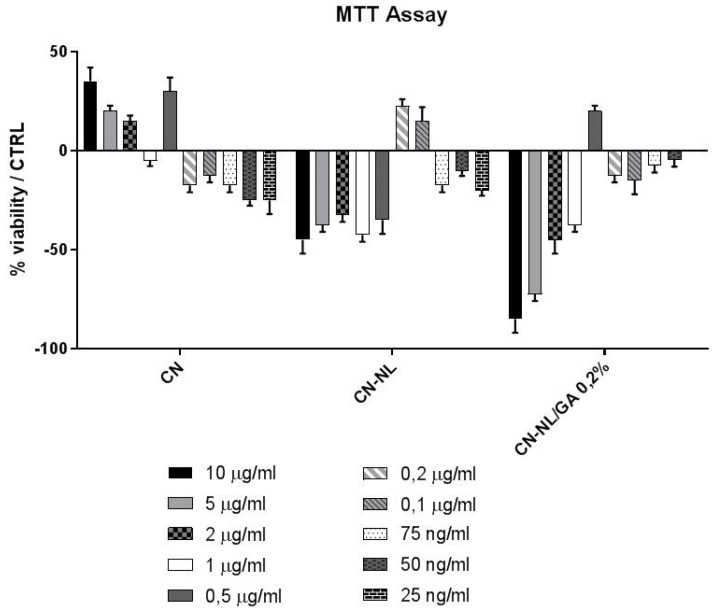
Results of MTT assay for different concentrations of CN, CN-NL, CN-NL/GA (0.2%), using HaCaT cells. The results were normalized by the viability of untreated cells as control.

After 24 h, the cells were incubated with MTT at 0.5 mg/mL at 37 °C for four 4 h and, subsequently, with DMSO at room temperature for 15 min. The spectrophotometric absorbance of the samples was determined by using an Ultra Multifunctional Microplate Reader (Biorad) at 570–655 nm [21]. Results, expressed as percentage of viable treated cell to respect with untreated control, are shown in Figure 6. In vitro studies performed on HaCaT cells enabled the establishment of the safe concentration of these green nano- and micro-compounds to be used. In particular, CN and CN-NL, did not decrease cell viability at 10 µg/mL and 0.2 µg/mL, respectively. Furthermore, CN-NL/GA complexes did not decrease cell viability at 0.5 µg/mL.

### 2.4. HMSC Morphology, Viability and Osteodifferentiative Potential

**Figure 7:**
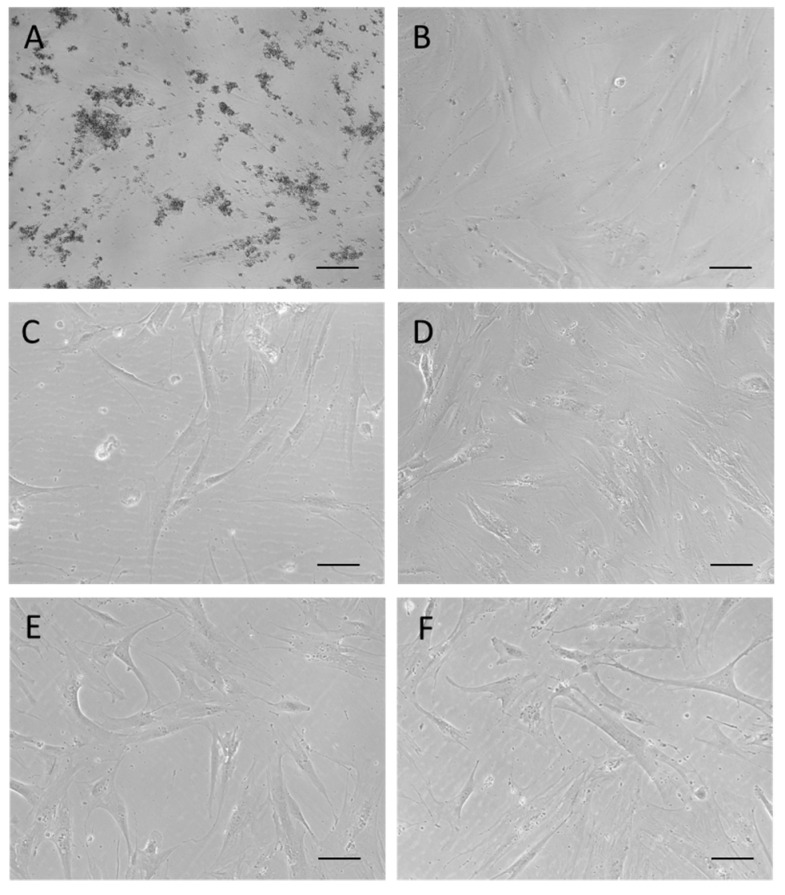
Micrographs at the inverted optical microscope of hMSCs in culture after 24 h of treatment with: (**A**) CN at 10 µg/mL; (**B**) NL at 10 µg/mL; (**C**) CN-NL at 0.2 µg/mL; (**D**) CN-NL/GA at 0.5 µg/mL; (**E**) GA at 0.5 µg/mL; (**F**) control. Micrographs are representative of each compound administrated at the intermediate concentration in the tested ranges. Original magnification 100×, scale bar = 100 µm.

**Figure 8:**
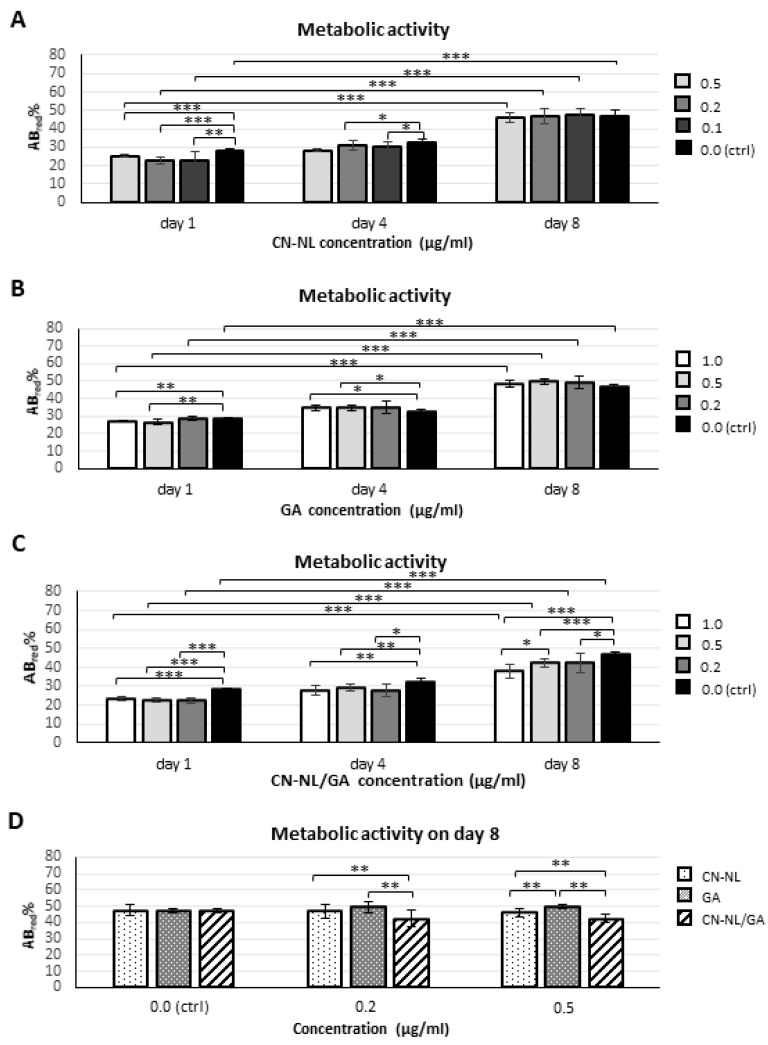
Bar graphs showing metabolic activity, as obtained by alamarBlue® test, performed on undifferentiated hMSCS using CN-NL, GA and CN-LN/GA complexes: (**A**–**C**) at different time points (1, 4 and 8 days in culture) and selected concentrations for each compound; (**D**) on day 8, comparing the three compounds within homogeneous concentration groups (* *p* < 0.01, ** *p* < 0.001, *** *p* < 0.0001).

Basing on the findings reported in Figure 6, hMSCs were treated with the above reported concentrations employed for HaCaT cells and the cell culture results are shown in Figure 7 and Figure 8.

Lower and higher concentrations of CN-NL (0.1 µg/mL and 0.5 µg/mL) and both CN-NL/GA and GA alone (0.2 µg/mL and 1 µg/mL) were tested with hMSCs. CN and NL were assayed at the maximum concentration. Treatments with CN, LN, CN-LN, CN-LN/GA complexes and GA did not affect the morphology of hMSCs at all the tested concentrations (Figure 7). CN treated samples showed micrometric aggregations that did not alter cell adhesion and growth (Figure 7A). Beside using pure CN, the other samples appeared clean and the spindle-like shape of hMSCs was clearly visible. AlamarBlue® test was performed on undifferentiated hMSCs in growth conditions in order to assess the cytocompatibility of the CN-NL complex and GA separately, and the combined CN-NL/GA complex. The obtained results are shown in Figure 8. 

**Figure 9:**
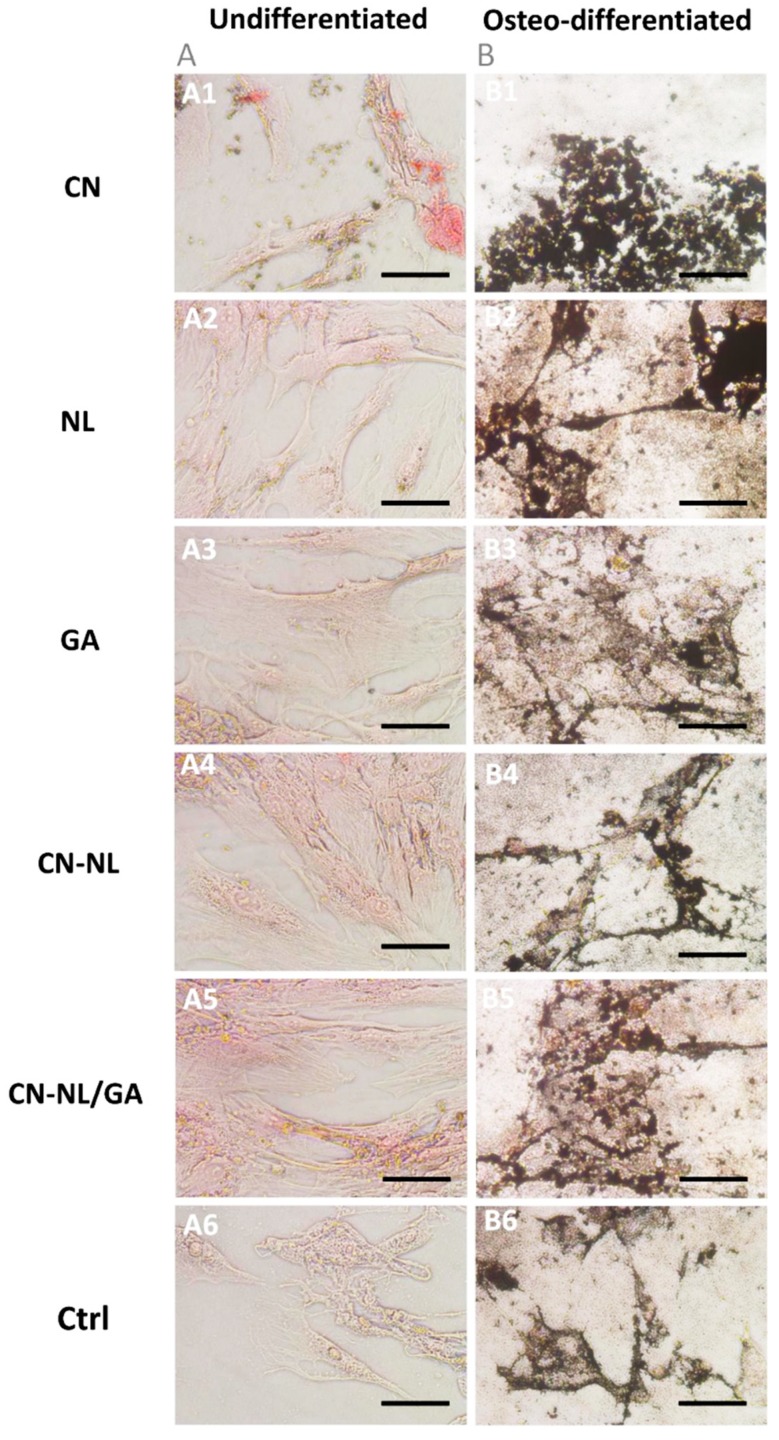
Light micrographs of von Kossa staining performed on (**A**) undifferentiated (control) and (**B**) osteodifferentiated hMSCs after incubation with all the nanocomponents (CN, NL, GA, CN-NL, CN-NL/GA) for 15 days at the highest concentrations in the tested ranges. Original magnification 200×; scale bar = 100 µm.

In all the samples, the metabolic activity significantly increased over time (*p* < 0.0001) and was not significantly different from that of control samples (*p* = n.s), but using CN-NL/GA. In these samples, at the endpoint, the metabolic activity statistically decreased with respect to controls by increasing CN-NL/GA concentration (*p* < 0.01 at 0.2 µg/mL; *p* < 0.0001 at 0.5 µg/mL and 1.0 µg/mL) (Figure 8C). The highest GA concentrations (0.5 µg/mL and 1.0 µg/mL) increased hMSC metabolic activity with statistical significance only at day 4 (*p* < 0.01) (Figure 8B). On day 8, GA resulted the most performing treatment over CN-NL and CN-NL/GA both at 0.2 µg/mL and 0.5 µg/mL; instead, CN-NL/GA reduced hMSC metabolic activity (*p* < 0.001) (Figure 8D). To assess if the multipotency of hMSCs was maintained by the administration of these green compounds, the hMSCs were differentiated towards the osteogenic lineage in presence of all the additives and compared to undifferentiated hMSCs as a control. Mineral matrix deposition was investigated as a proof of osteo-differentiation using von Kossa staining (Figure 9). 

The obtained findings highlighted that both treated and untreated hMSCs, cultured in osteodifferentiation medium, produced mineral granules, visible in black, with no appreciable differences according to morphology and extension (Figure 9). 

### 2.5. Anti-Inflammatory and Immune Responses of HaCaT Cells

**Figure 10:**
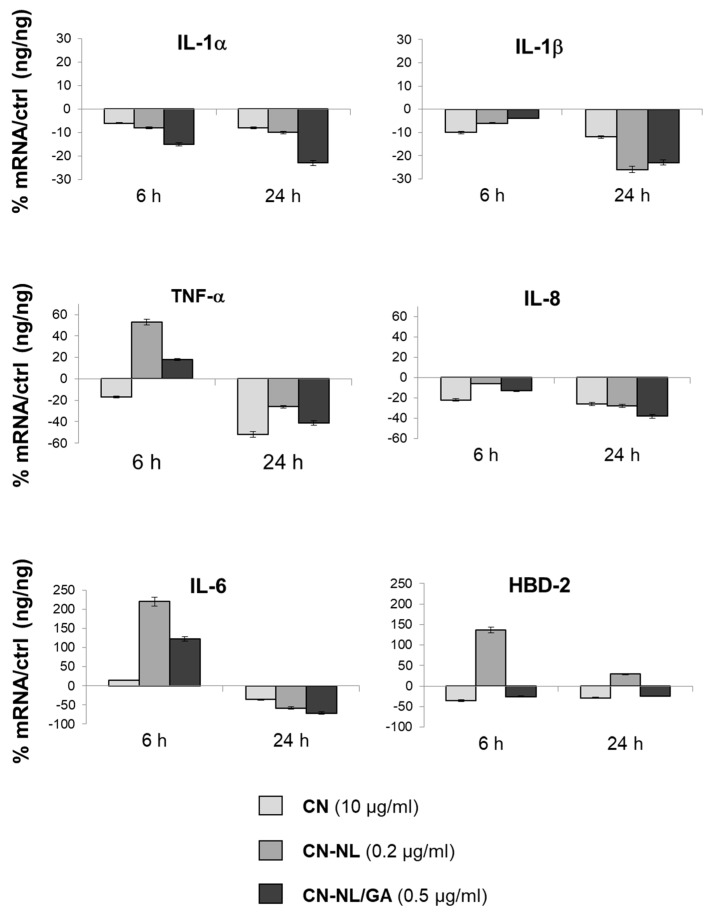
Bar graphs showing the results of RT-PCR performed on HaCaT cells exposed to the biopolymer nanomaterials al 6 h and 24 h for different cytokines involved in inflammatory response and HBD-2 and an antimicrobial peptide. The results are normalized by the expression in untreated cells as control.

Our data indicated that CN, CN-NL and GA markedly down-regulated the expression of pro-inflammatory cytokines interleukin-1 alpha (IL-1α), IL-1β, IL-6, IL-8 and tumor necrosis factor alpha (TNF-α) at mRNA level, especially after 24 h of treatment. The observed panel is in line with modulation of inflammatory activity exerted by all these components on human keratinocytes (Figure 10). The CN-NL/GA complex was the most effective in modulating the expression of the largest number of pro-inflammatory cytokines: IL-1α, IL-6, and IL-8. CN-NL was able to up-regulate the expression of an antimicrobial peptide, the human β-defensin (HBD-2), which is involved in innate immune response (Figure 10). Differently, transforming growth factor beta (TGF-β) was not modulated (data not shown). 

These results are very encouraging for the use of these natural products for cosmetic applications and tissue repair, by acting on the modulation of cytokines and antimicrobial peptide HBD-2. On the other hand, these composites did not modify the proliferative and differentiative potential of hMSCs, thus demonstrating a good biocompatibility and cell affinity. 

## 3. Discussion

The spray-dried CN-NL complex in powder was found to be water-suspendable and applicable on a surface in the form of micrometric flat nanostructured ellipsoids. Regulating the concentration of the water suspension can be fundamental to treat and confer to a surface some functional properties, such as anti-microbial and antioxidant properties. The resultant NL thermal stability was lower than that of CN. However, the CN-NL complex displayed an improved thermal stability with respect to pure NL. In the presence of GA, despite of its low amount in the powder (2% by weight), the thermal stability was further improved, in agreement with the radical scavenging properties of this substance. On the whole the complex CN-NL in powder, also in its version loaded with GA, can be considered a functionalizing agent much useful for functionalizing various substrates. 

Interaction with skin cells is fundamental to assess the functional properties and demonstrate safety in skin contact applications, as they may include cosmetics, personal care as well as skin regeneration. It is a fact that nanosized objects can migrate into tissues [7], potentially leading to unknown reactions. The nanomaterials investigated in this study are biodegradable and therefore their interaction with the human body is temporary, so they do not pose harmful risks due to organ accumulation. The initial interactions with skin cells was investigated to understand their safety and disclose possible inflammatory reactions. In our study, we used HaCaT cells, as a model of human keratinocytes (i.e., epithelial cells), to select the optimal concentrations to be used for each of these biocompounds, which were 10 µg/mL for CN and NL alone, 0.2 µg/mL for CN-NL and 0.5 µg/mL for CN-NL/GA complexes. The higher concentration usable for CN and NL in comparison with those of the complexes may depend on the larger size (micrometric) of the CN-NL. 

HMSCs were used as a model of mesenchymal (stem) cells present in the innermost layers of the skin, such as dermis and hypodermis (where they originate fibroblasts, among other cell types), as well as connective tissue in general [24], in order to assess the cytocompatibility and safety. Starting from the optimal concentrations chosen for HaCaT cells, each component was diluted at higher and lower concentrations to investigate the sensitivity of hMSCs to the same component. In the selected concentration ranges, the hMSCs were viable, with no appreciable differences in their morphology and osteo-differentiation capability with respect to untreated samples [25], thus demonstrating good biocompatibility and cell affinity of these green components, even at two times higher concentrations than the optimal ones selected using HaCaT cells. The wide range of cytocompatibility with hMSCs ensures a safe use of these bionanomaterials in the selected ranges. The significant reduction in hMSC metabolic activity following the administration of CN-NL/GA samples highlighted at the endpoint (day 8) with respect to GA and CN-LN administrated singularly can be a consequence of the synergistic activity exerted by the two components in the complex, and in particular by CN-NL, which alone largely decreased the metabolic activity of HaCaT cells at 1 µg/mL concentration. GA is known to affect some cellular functions in hepatocytes [17], and in our study showed a positive effect on hMSC metabolism. It is thus possible that the size and the neutral charge of the complex can affect cell metabolic activity by promoting other cellular functions. 

HaCaT cells were also used to understand the reaction of the epidermis layer by investigating an array of cytokines involved in inflammation and immune response. HBD-2 is an inducible antimicrobial peptide with a molecular mass of 4–6 kDa acting as an endogenous antibiotic in the defense of host against Gram-positive and Gram-negative bacteria, fungi and the envelope of some viruses, and is involved in the innate immune response because its release is induced by pro-inflammatory cytokines, endogenous stimuli, infections or wounds [26]. Cytokines are multi-functional biological molecules that play a key role in the innate immune response, and that are involved in important biological activities such as hematopoiesis, infectious diseases, tumorigenesis, homeostasis and tissue repair, growth and cell development [27]. In particular, IL-1 promotes local inflammation and coagulation, increases the expression of adhesion molecules, and causes the release of chemokines and recruitment of leukocytes to the site of inflammation. In addition, it is an endogenous pyrogen, stimulates the synthesis of acute phase proteins and induces cachexia [28]. TNF-α is essential mediator in inflammation [29]. Its release in the site of the inflammation involves a localized vascular endothelial activation and vasodilation with increased vascular permeability. TNF-α also acts on platelet adhesiveness, favoring the formation of thrombus and occlusion of blood vessels and, therefore, reducing infection but also the tissue necrosis. IL-6 is a pleiotropic molecule that stimulates hepatocytes to synthetize many plasma proteins, such as fibrinogen, which ultimately contribute to the inflammatory acute phase response. Finally, IL-8 is a chemokine with many functions, including the attraction and activation of polymorphonuclear leukocytes, chemotaxis of basophils and a role in angiogenesis [30]. Remarkably, CN-NL and CN-NL/GA upregulated the mRNA expression of TNF-α and IL-6 at 6 h, which resulted subsequently downregulated after 24 h. This behavior can be explained by giving the nanocomposites a role in the wound repair process. In fact, wound healing is characterized a series of phases, and in particular in a first phase a number of overlapping events occur, including the production of pro-inflammatory cytokines. TNF-α represents the primary cytokine for pro-inflammatory responses; the direct effect of its release is the upregulation of IL-6 [31]. The obtained results showed that all the selected components were able to modulate the inflammatory response by decreasing the expression of proinflammatory cytokines. This fact was particularly evident in the CN-NL/GA complex, which downregulated the mRNA expression of the largest number of pro-inflammatory cytokines tested: IL-1α, IL-6, and IL-8. The unloaded complex, CN-NL alone, was able to up-regulate the expression of HBD-2. The reason why CN-NL/GA does not maintain the same functional activity towards HBD-2 may depend on specific cellular interactions with the GA-loaded complex. Indeed, GA shows different antibacterial mechanisms that are exerted directly towards bacteria and are not cell-mediated [32]. The obtained findings showed that CN, NL and specifically CN-NL complexes are very promising for skin contact applications, such as for coating biomedical, personal care and cosmetic products. Further investigation using primary human keratinocytes will provide a comprehensive validation of these compounds. 

## 4. Materials and Methods 

### 4.1. Materials

CN, NL, and GA were supplied by Mavi Sud, Aprilia (LT), (Milan, Italy). CN, CN-NL and CN-NL/GA were prepared in powder by using a Buchi Mini B-190 spray drier (Flawil, Switzerland) [33] by adding 2% with respect to CN-NL of PEG8000 from Sigma-Aldrich (Milan, Italy). Complexes CN-NL and CN-NL/GA were thus prepared in accordance with previous works [34,35]. The ratio between CN and NL is 2:1 by weight. The content of GA in CN-NL/GA is 0.2% by weight. HMSCs were supplied from Merck Millipore S.A.S., (Burlington, MA, USA). HaCaT, Dulbecco’s Minimal Essential Medium (DMEM), L-glutamine, penicillin, streptomycin and fetal calf serum were purchased from Invitrogen, (Carlsbad, CA, USA). Fetal Bovine Serum and AlamarBlue® were purchased from Thermo Fisher Scientific, (Waltham, MA, USA). The HMSC Differentiation Osteogenic Bulletkit was purchased from Lonza, (Basel, Switzerland). Dulbecco’s phosphate-buffered saline (DPBS), silver nitrate, pyrogallol, sodium thiosulphate, nuclear fast red, dimethyl sulfoxide (DMSO), MgCl_2_ and MTT were purchased from Sigma-Aldrich (Milan, Italy). Aluminum sulphate was purchased from Carlo Erba (Milan, Italy). 

### 4.2. Morphological Characterization of CN, NL and CN-NL Complexes

The morphology of the materials samples was investigated by field emission scanning electron microscopy (FESEM) using a FEI FEG-Quanta 450 instrument (Field Electron and Ion Company, Hillsboro, OR, USA). The samples were sputtered with Gold (Gold Edwards SP150B, England) before analysis. Inverted optical microscope (Nikon Ti, Nikon Instruments, Amsterdam, The Netherlands) was used to evaluate the morphology of hMSCs on CN, NL and CN-NL and CN-NL/GA complexes. 

### 4.3. Chemical Structure and Thermal Stability Charachterisation of CN, NL and CN-NL Complexes

The powders as provided by MAVI were characterized by infrared spectroscopy using a Nicolet T380 Thermo Scientific instrument equipped with a Smart ITX ATR accessory with diamond plate. 

Thermogravimetric tests onto the powders were performed on 4–10 mg of sample using a Mettler-Toledo Thermogravimetric Analysis/Scanning Differential Thermal Analysis (TGA/SDTA) 851 instrument operating with nitrogen as the purge gas (60 mL/min) at 10 °C/min heating rate in the 25–800 °C temperature range.

### 4.4. In vitro Culture of hMSCs and HaCaT

CN, NL, CN-NL, 0.2%GA and CN-NL/0.2%GA were solubilized in DPBS at 0.1 mg/mL and then diluted in the culture media to be tested with the cells. HMSCs isolated from the bone marrow were cultured in 24 well plate at 20,000 cells/well in DMEM Low Glucose with 2 mM L-glutamine, 100 IU/mL penicillin, 100 mg/mL streptomycin and 10% heat-inactivated FBS, with the above-mentioned components at different concentrations for 1 week to select the best concentration to be used. Immortalized human keratinocytes (HaCaT cell line) were cultured in DMEM supplemented with 1% Pen-Strep, 1% glutamine and 10% fetal calf serum at 37 °C in air and 5% CO_2_. Subsequently, cells were dispensed into 6-well culture plates and left to grow until 80% of confluence. 

### 4.5. MTT Assay

To establish the optimal non-toxic concentrations to be used in subsequent treatments, HaCaT cells were seeded at a density of 1 × 10^3^/well in 96-well culture plates (*n* = 2). After 24 h, the cells were treated with CN, CN-NL and CN-NL/GA at different concentrations (from 10 µg/mL to 25 ng/mL) for 24 h and then incubated with MTT (0.5 mg/mL) at 37 °C for 4 h and, subsequently, with DMSO at room temperature for 5 min. The spectrophotometric absorbance of the samples was determined by using Ultra Multifunctional Microplate Reader (Bio-Rad, Hercules, California, USA) at 570–655 nm [36]. Results were given as average in Table 2.

### 4.6. Evaluation of hMSCs Viability and Differentiation Potential

AlamarBlue® test was performed at days 1, 4 and 8 to monitor hMSC viability in 24 well plates (*n* = 6) following the manufacturer’s instructions. HMSCs were cultured for one week with the following concentrations of the components (Table 2):

Subsequently, hMSCs were osteoinduced using the differentiation Bulletkit osteogenic medium for one week in order to test hMSC regeneration potential. The samples plates (*n* = 3) were fixed with 1% *w*/*v* neutral buffered formalin. Mineralized matrix production was detected by von Kossa staining: cells were incubated with 1% *w*/*v* silver nitrate exposed to light for 15 min, 0.5% *w*/*v* Pyrogallol for 2 minutes and 5% *w*/*v* sodium thiosulphate for 2 min. All the solutions were in distilled water. The counterstaining was performed incubating cells with 0.1% *w*/*v* nuclear fast red diluted in a distilled water solution containing 5% *w*/*v* aluminum sulphate, for 5 min and washing in tap water for 5 min in order to reveal the reaction. The staining was observed with a Nikon Eclipse Ci microscope (Nikon Instruments, Amsterdam, The Netherlands) equipped with a digital camera by three independent observers for a qualitative analysis. 

### 4.7. Anti-Inflammatory and Immune Responses Evaluation of HaCaT Cells

HaCaT cells, seeded in 6-well culture plates (*n* = 3) until 80% of confluence, were treated with CN, CN-NL and CN-NL/GA at selected concentrations (i.e., 10 µg/mL, 0.2 µg/mL and 0.5 µg/mL, respectively) for 6 h and 24 h. At the end of the experiment, total ribonucleic acid (RNA) was isolated and one microgram of this were reverse-transcribed into complementary deoxyribonucleic acid (cDNA) using random hexamer primers (Promega, Italy) at 42 °C for 45 min, according to the manufacturer’s instructions. The anti-inflammatory and immune responses of HaCaT cells were evaluated by assaying the expression of pro-inflammatory cytokines IL-1α, IL-1β, IL-6, IL-8 and TNF-α, anti-inflammatory cytokine TGF-β, and antimicrobial peptide HBD-2 by Real time PCR with the LC Fast Start DNA Master SYBR Green kit from Roche Applied Science (Euroclone S.p.A., Pero, Italy) using 2 µl cDNA, corresponding to 10 ng of total RNA in a 20 Mm µl final volume, 3 mM Magnesium Chloride (MgCl_2_) and 0.5 µM of sense primer and antisense primers (Table 3).

At the end of each run, the melting curve profiles were achieved by cooling the sample to 65° C for 15 s and then heating it slowly at 0.20 °C/s up to 95 °C with continuous measurement of fluorescence to confirm the amplification of specific transcripts. Cycle-to-cycle fluorescence emission readings were monitored and analyzed using LightCycler® software (Roche Diagnostics GmbH). Melting curves were generated after each run to confirm the amplification of specific transcripts. We used the b-actin coding gene, one of the most commonly used housekeeping genes, as an internal control gene. All reactions were carried out in triplicate, and the relative expression of a specific mRNA was determined by calculating the fold change relative to the b-actin control. The fold change of the tested gene mRNA was obtained with LightCycler® software by using the amplification efficiency of each primer, as calculated by the dilution curve. The specificity of the amplification products was verified by subjecting the amplification products to electrophoresis on 1.5% agarose gel and visualization by ethidium bromide staining [37].

### 4.8. Statistical Analysis

Statistical analyses were carried out by SPSS (SPSS v.16.0; IBM). All data were analyzed using a one-way analysis of variance (ANOVA) and post hoc test (Duncan) for multiple comparisons. Probability (*p*) values < 0.05 were considered as statistically significant differences.

## 5. Conclusions

CN-NL and CN-NL containing GA, obtained in powder by a spray drier technology can be optimal agents to form nanostructured and functional surfaces through a simple deposition by water suspension. The thermal stability of the complexes CN-NL and CN-NL/GA were investigated, and it was found that they can be considered thermally stable up to 114 °C and 127 °C, respectively. The performed characterization can give indications for a suitable application of CN-NL complexes as coating for material surfaces. All the investigated biopolymeric components were cytocompatible with HaCaT cells and hMSCs and optimal concentrations were selected for each of them. These findings demonstrate that CN-NL/GA complexes are able to downregulate a panel of anti-inflammatory cytokines in human keratinocytes and do not modify the proliferative and osteo-differentiative capacity of hMSC. Thus, these materials are very promising for skin contact applications, such as in biomedical, personal care and cosmetic products.

## Figures and Tables

**Table 1:** Results of TGA analysis.

	Onset 1 (°C)	Mass Loss 1 (%)	Onset 2 (°C)	Mass Loss 2 (%)	Onset 3 (°C)	Mass Loss 3 (%)	Residue (%)
GA	386.95	−98.92	-	-	-	-	1.97
NL	40	−5.26	262	−55.63	-	-	38.93
CN	159	−5.35	300	−57. 54	387	−17.85	17.42
CN-NL	114	−2.60	266	−69.25	406	−10.39	12.37
CN-NL/GA	127	−1.92	251	−68.53	472	−11.63	13.84

CN, CN-NL and CN-NL/GA represent the spray-dried samples containing 2% (*w*/*w*) of PEG.

**Table 2:** Concentrations of compounds tested with hMSCs.

**CN-NL**	0.5 µg/mL	0.2 µg/mL	0.1 µg/mL
**GA**	1 µg/mL	0.5 µg/mL	0.2 µg/mL
**CN-NL/GA**	1 µg/mL	0.5 µg/mL	0.2 µg/mL

**Table 3:** Primer sequences and RT-PCR conditions.

Gene	Primers Sequence	Conditions	Product Size (bp)
IL-1α	5’-CATGTCAAATTTCACTGCTTCATCC -3’ 5’-GTCTCTGAATCAGAAATCCTTCTATC -3’	5 s at 95 °C, 8 s at 55 °C, 17 s at 72 °C for 45 cycles	421
TNF-α	5’-CAGAGGGAAGAGTTCCCCAG -3’ 5’-CCTTGGTCTGGTAGGAGACG -3’	5 s at 95°C, 6 s at 57°C, 13 s at 72°C for 40 cycles	324
IL-6	5’-ATGAACTCCTTCTCCACAAGCGC-3’ 5’-GAAGAGCCCTCAGGCTGGACTG-3’	5 s at 95°C, 13 s at 56°C, 25 s at 72°C for 40 cycles	628
IL-8	5-ATGACTTCCAAGCTGGCCGTG -3’ 5-TGAATTCTCAGCCCTCTTCAAAAACTTCTC-3’	5 s at 94°C, 6 s at 55°C, 12 s at 72°C for 40 cycles	297
TGF-β	5’-CCGACTACTACGCCAAGGAGGTCAC-3’ 5’-AGGCCGGTTCATGCCATGAATGGTG-3’	5 s at 94°C, 9 s at 60°C, 18 s at 72°C for 40 cycles	439
IL-1β	5’-GCATCCAGCTACGAATCTCC-3’ 5’-CCACATTCAGCACAGGACTC-3’	5 s at 95°C, 14 s at 58°C, 28 s at 72°C for 40 cycles	708

## Abbreviations

CN	Chitin nanofibrlis
NL	Nanolignin
GA	Glycyrrhetinic acid
HaCaT	Human keratinocytes
HMSCs	Human mesenchymal stromal cells
IL	Interleukin
TNF-α	Tumor necrosis factor alpha
PEG	Poly(ethylene glycol)
HBD-2	Human beta-defensin 2
TGF-β	Transforming growth factor beta
DMEM	Dulbecco’s Minimal Essential Medium
DPBS	Dulbecco’s phosphate-buffered saline
RT-PCR	Reverse transcription polymerase chain reaction
DMSO	Dimethyl sulfoxide
RNA	Ribonucleic acid
cDNA	Complementary deoxyribonucleic acid
MgCl2	Magnesium chloride
FESEM	Field Emission Scanning Electron Microscopy
ATR	Attenuated Total Reflection
TGA/SDTA	Thermogravimetric Analysis/Scanning Differential Thermal Analysis
